# Prognostic relevance of prognostic nutritional indices in gastric or gastro-esophageal junction cancer patients receiving immune checkpoint inhibitors: a systematic review and meta-analysis

**DOI:** 10.3389/fimmu.2024.1382417

**Published:** 2024-06-20

**Authors:** Shufu Hou, Dandan Song, Ruiqi Hao, Linchuan Li, Yun Zhang, Jiankang Zhu

**Affiliations:** ^1^ Department of General Surgery, The First Affiliated Hospital of Shandong First Medical University, Jinan, China; ^2^ Key Laboratory of Metabolism and Gastrointestinal Tumor, The First Affiliated Hospital of Shandong First Medical University, Jinan, China; ^3^ Department of Neurology, Shandong Province Third Hospital, Jinan, China

**Keywords:** gastric cancer, immune checkpoint inhibitors, gastro-esophageal junction cancer, prognostic nutritional index, meta – analysis

## Abstract

**Background:**

The Prognostic Nutritional Index (PNI) has become an important predictive tool for assessing patients’ nutritional status and immune competence. It is widely used in prognostic evaluations for various cancer patients. However, the prognostic relevance of the Prognostic Nutritional Index (PNI) in gastric or gastro-esophageal junction cancer patients (GC/GEJC) undergoing immune checkpoint inhibitors (ICIs) treatment remains unclear. This meta-analysis aimed to determine the prognostic impact of PNI in this specific patient cohort.

**Methods:**

We conducted a thorough literature search, covering prominent databases such as PubMed, Embase, Web of Science, SpringerLink, and the Cochrane Library. The search spanned from the inception of these databases up to December 5, 2023. Employing the 95% confidence interval and Hazard Ratio (HR), the study systematically evaluated the relationship between PNI and key prognostic indicators, including the objective remission rate (ORR), disease control rate (DCR), overall survival (OS) and progression-free survival (PFS) in GC/GEJC patients undergoing ICI treatment.

**Results:**

Eight studies comprising 813 eligible patients were selected. With 7 studies consistently demonstrating superior Overall Survival (OS) in the high-Prognostic Nutritional Index (PNI) group compared to their low-PNI counterparts (HR 0.58, 95% CI: 0.47–0.71, P<0.001). Furthermore, the results derived from 6 studies pointed out that the significant correlation between he low-PNI and poorer progression-free survival (PFS) (HR 0.58, 95% CI: 0.47–0.71, P<0.001). Subgroup analyses were performed to validate the robustness of the results. In addition, we conducted a meta-analysis of three studies examining the correlation between PNI and objective response rate/disease control rate (ORR/DCR) and found that the ORR/DCR was significantly superior in the high PNI group (ORR: RR: 1.24, P=0.002; DCR: RR: 1.43, P=0.008).

**Conclusion:**

This meta-analysis indicates that the low-PNI in GC/GEJC patients undergoing ICI treatment is significantly linked to worse OS and PFS. Therefore, PNI can serve as a prognostic indicator of post-treatment outcomes in patients with GC receiving ICIs. Further prospective studies are required to assess the reliability of these findings.

**Systematic review registration:**

https://inplasy.com/, identifier INPLASY202450133.

## 1 Introduction

Gastric cancer is a malignant tumor that can aggressively invade the gastrointestinal tract and metastasize to distant sites. Globally, it ranks as the fifth most common cancer and the fourth leading cause of cancer-related mortality (1, 2). Although the incidence and mortality rates of gastric and gastroesophageal junction cancers have declined in recent years, these cancers still pose significant challenges and threats to human health (3). With changes in lifestyle habits, the incidence rate among younger populations is on the rise (4). Gastric and gastroesophageal junction cancers are characterized by insidious onset, rapid progression, high malignancy, and poor prognosis (5). The majority of patients diagnosed with gastric or gastroesophageal junction cancer are typically already in advanced stages of the disease at the time of diagnosis. As a result, the benefits of surgery are much lower compared to those for early-stage patients, and some may even lose the opportunity for curative surgery. In recent years, significant progress has been made in treating gastric and gastroesophageal junction cancers through combined chemotherapy and immunotherapy, particularly with the use of immune checkpoint inhibitors (ICIs) for advanced-stage cases (6). The combination of chemotherapy with immunotherapy and the integration of immune checkpoint inhibitors with other treatments have been continuously emerging (7). For example, The concurrent use of PD-1 and CTLA-4 inhibitors targets distinct stages of T cell activation., thereby producing a synergistic effect (8). Immune checkpoint inhibitors (ICIs) have become a mainstream treatment for various malignant tumors and have revolutionized traditional cancer therapy (9–13). ICIs have demonstrated significant advantages in improving the survival rates of patients with gastric or gastroesophageal junction cancer (14–17). In recent years, despite the improvement in prognosis for gastric or gastroesophageal junction cancer through the combination of chemotherapy and immune checkpoint inhibitors (ICIs), there has been an increasing incidence of immune-related adverse events (irAEs) associated with their use. Particularly, anti-CTLA-4 therapy appears to induce more severe irAEs compared to anti-PD-1/PD-L1 treatments, affecting multiple organ systems, and is more common in patients who respond well to treatment (18). As the approval of ICIs rapidly expands across various cancer types, knowledge regarding risk factors and biomarkers can help providers assess the risk of immune-related adverse events (irAEs) in patients. For instance, biomarkers such as cytokines (19), HLA (20), autoantibodies (21), gene expression profiles (22), among others, can aid in assessing the risk of irAEs. However, the above-mentioned biomarkers are costly and involve complex procedures. Additionally, tumor mutational burden (TMB) (23), tumor-infiltrating lymphocytes (24), microsatellite instability (MSI) (25), and other tumor biomarkers have been extensively studied as predictive biomarkers for PD-1/L1 inhibitor therapy. However, due to the relatively complex detection processes and a lack of consensus on numerical thresholds, their clinical application has been limited. Malnutrition accounts for 87% of gastric cancer patients, and the incidence of cachexia is as high as 65%-85%, exceeding all other tumors, and both malnutrition and cachexia incidence account for the first place of all tumors (26). The Prognostic Nutritional Index (PNI), initially proposed by the Buzby team (27), is used to assess overall nutritional and immune status. It quantifies serum albumin and peripheral blood lymphocyte count through a simple calculation. Clinicians can predict the risk of postoperative complications in surgical patients by assessing preoperative nutritional status (28). Evaluating the nutritional and immune status of the body holds significant clinical importance in predicting the prognosis of cancer patients (29–31). The mechanical obstruction and progression of gastric or gastroesophageal junction cancer deteriorate patients’ nutritional status, affecting serum albumin levels and impairing the host’s immune status (32). Additionally, lymphocytes, which are targeted by ICIs, inhibit tumors and play a crucial role in tumor immunity. Consequently, lymphocyte count is widely used as an indicator of immune competence (33). However, there is currently a lack of meta-analyses on the predictive significance of PNI in patients with gastric or gastroesophageal junction cancer undergoing ICI therapy. Therefore, we included relevant cohort studies to compare the prognosis and treatment response among different PNI groups of these patients after ICI treatment. This study aims to explore the prognostic value of PNI in this patient cohort.

## 2 Materials and methods

### 2.1 Search strategy

This meta-analysis strictly followed the guidelines specified in the Preferred Reporting Items for Systematic Reviews and Meta-Analyses (PRISMA) checklist (34). The PRISMA checklist ensures a comprehensive and transparent reporting of systematic reviews and meta-analyses, emphasizing methodological clarity and quality. The study protocol has been registered with the International Platform of Registered Systematic Review and Meta-analysis Protocols (INPLASY)(Registration ID: INPLASY202450133). Thorough searches were conducted by two independent researchers, encompassing multiple databases, namely PubMed, Embase, Web of Science, SpringerLink, and the Cochrane Library. The search duration spanned from the establishment of these databases until December 13, 2023. It utilizes the following terms to investigate the predictive significance of PNI and ICIs in patients with gastric or gastro-esophageal junction cancer: “Prognostic Nutritional Index” or “PNI “ and “gastric” or “stomach” or “esophagogastric junction “or “esophageal” or “oesophageal” or “esophagus” or “esophageal” and “cancer” or “tumor “ or “carcinoma “ or “adenocarcinoma “ or “neoplasm” and “PD-L1 inhibitors” or “immune checkpoint inhibitors” or “programmed cell death ligand-1 inhibitors” or “immunotherapy” or “ICIs”. In addition to utilizing free search terms and Medical Subject Headings (MeSH) for searching within titles or abstracts, we screened the references of selected articles to ensure comprehensive retrieval.

### 2.2 Inclusion and exclusion criteria

Inclusion criteria: (1) The patient was diagnosed with GC/GEJC through a comprehensive evaluation, which included imaging studies, serum tumor marker tests, and a histopathological biopsy; (2) Received ICIs, either in combination with chemotherapy or as a stand-alone drug; (3) Provided survival data in the distant future such as overall survival (OS) or progression-free survival (PFS), and the existence of feedback of therapeutic data such as the objective remission rate (ORR) or the disease control rate (DCR); (4) Data such as HR and 95% CI can be obtained in the literature directly or indirectly;(5)The methods of study were either cohort study or randomized controlled study.

Exclusion criteria: (1) reviews, case reports, case series, conference abstracts, or commentaries; (2) data overlap or duplication; (3) the literature fails to provide complete raw data information.

### 2.3 Data extraction and quality assessment

Two researchers conducted independent literature searches, following predetermined criteria and specified strategies. This approach ensures a thorough and unbiased exploration of available literature, utilizing a systematic and structured methodology. Meticulous data extraction was performed, encompassing essential details such as the first author’s name, publication year, study country, design, sample size, gender distribution, treatment modalities, and survival analyses, including OS、PFS,、DCR、ORR. The quality of each study was meticulously evaluated using the Newcastle-Ottawa Scale (NOS) (35). Studies scoring above 6 points were considered high-quality indicators. This stringent evaluation ensures that only studies meeting robust methodological standards contribute to the overall analysis. Use the following equation to calculate the PNI value: 10 * serum albumin value + 0.005 * peripheral blood lymphocyte count. This standardized calculation method allows for consistent and comparable PNI values across the studies, enhancing the reliability and validity of the meta-analysis results.

### 2.4 Data statistics

Statistical analysis in this study was performed using Stata SE (version 12.0; StataCorp, College Station, Texas, USA). Heterogeneity among the studies was evaluated using Cochran’s Q-test and I2 statistics. In cases where heterogeneity was not significant (P≥0.10 or I²<50%), a fixed-effects model was applied; conversely, in the presence of significant heterogeneity (P<0.10 or I²≥50%), a random-effects model was employed for the meta-analysis. Effect sizes for dichotomous variables, such as ORR and DCR, were represented using RR along with their 95% CI. For survival data, including OS and PFS, Hazard Ratios (HR) and their 95% CI were utilized. The threshold for statistical significance was defined as P < 0.05. To evaluate publication bias, we examined the symmetry of the funnel plot and employed methods such as Egger’s linear regression and Begg’s regression, with a P-value < 0.05 indicating potential publication bias. A sensitivity analysis was conducted to assess the influence of individual studies on overall survival (OS) and progression-free survival (PFS).To explore the origins of heterogeneity, subgroup analyses were conducted, considering treatment modalities, sample sizes, cutoff values, and analytical models.

## 3 Results

### 3.1 Study selection and characteristics

**Figure 1:**
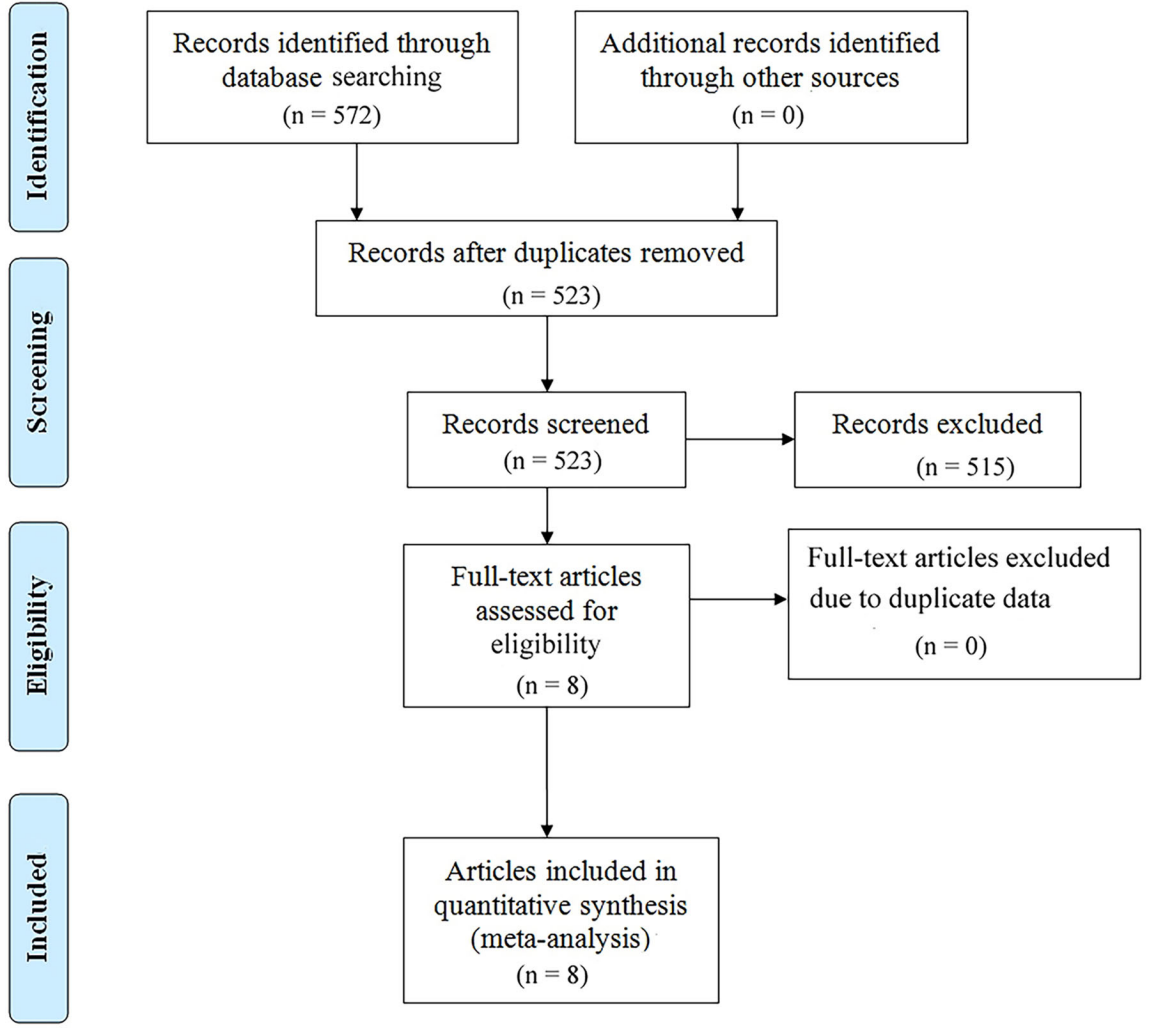
Prisma flowchart illustrating the literature selection process.

We searched PubMed, Embase, and Web of Science SpringerLink as well as Cochrane Library databases to find a total of 572 relevant papers, firstly, Based on the inclusion and exclusion criteria, 49 duplicates were removed from the dataset and then 515 papers were excluded by reviewing their abstracts, titles and other abbreviations, and finally, 8 articles were included after full-text review (36–43), These were retrospective studies with a total of 813 patients receiving ICIs. articles, These were retrospective studies, and a total of 813 patients with (GC/GEJC) treated with ICIs were included, and the flow chart is shown in **Figure 1**. **Table 1** provides a summary of the characteristics of the included studies. All of these studies were published between 2020 and 2023, including three from China, three from Japan, one from the United States, and one from South Korea. All eight articles were retrospective studies. The sample size ranged from 29 to 268, totaling 813 patients. Five studies used ICIs alone and three studies used a combination of chemotherapy including ICIs. Two studies focused only on overall survival (OS), one study focused only on progression-free survival (PFS), and the remaining five studies documented both overall survival (OS) and PFS. According to the NOS rating sheet, all of the included studies scored between 6 and 8, indicating relatively high data quality. **Table 2** presents the Newcastle-Ottawa Quality Assessment Scale (NOS) scores for all articles included in the analysis.

**Table 1:** Baseline characteristics of included studies.

Study, year	Country	Duration	Study design	Sample size	Age	Gender (M/F)	Follow-up (months)	Treatment	Cut-off	Survival outcome	Analysis
Namikawa 2020	Japan	2017–2019	Retrospective	29	median: 71 (49–86)	19/10	median: 32	Nivolumab	31.1	OS, PFS	U, U
Watanabe 2021	Japan	2015–2019	Retrospective	110	NR	79/31	NR	Nivolumab	40	OS, PFS	M, M
Booka 2022	Japan	2017–2021	Retrospective	30	46–86	23/7	NR	ICIs	37.2	OS	U
Lee 2022	Korea	2017–2021	Retrospective	35	median: 54.5 (25.0–71.0)	19/16	median: 2.0 (0.3–13.5)	Nivolumab	40	OS, PFS	M, M
Morelli 2022	UK	2014–2018	Retrospective	57	median: 61 (29–85)	43/14	median: 27 (4–53)	ICIs	33	OS	M
Sun 2022	China	2016–2020	Retrospective	89	NR	NR	NR	ICIs	31.1	OS, PFS	U, U
Pan 2023	China	2014–2021	Retrospective	268	median: 59 (18–86)	199/69	NR	ICIs	43.44	OS, PFS	M, M
Wang 2023	China	2020–2021	Retrospective	195	NR	135/60	NR	ICIs	45.5	PFS	U

M, male; F, female; NR, not report; nivolumab, a Programmed-Death-1 (PD-1) inhibitor; ICIs, immune checkpoint inhibitors; OS, overall survival; PFS, progression-free survival; U, univariate; M, multivariate; NOS, Newcastle-Ottawa Scale.

**Table 2:** Newcastle-Ottawa Scale (NOS) for quality assessment.

Studies	Selection				Comparability	Outcome			Scores
	A	B	C	D	E	F	G	H	
Namikawa 2020	★	★	★	★	★	★	★	–	7
Watanabe 2021	★	★	★	★	★★	★	–	–	7
Booka 2022	★	★	★	★	★	★	–	–	6
Lee 2022	★	★	★	★	★★	★	★	–	8
Morelli 2022	★	★	★	★	★★	★	★	–	8
Sun 2022	★	★	★	★	★	★	–	–	6
Pan 2023	★	★	★	★	★★	★	–	–	7
Wang 2023	★	★	★	★	★	★	–	–	6

A study may receive a maximum of one star for each numbered item in the Selection and Outcome categories. A maximum of two stars may be given for Comparability, as directed by the NOS. One star represents one point.

### 3.2 Prognostic impact of PNI on PFS and OS in GC/GEJC patients treated with ICIs

**Figure 2:**
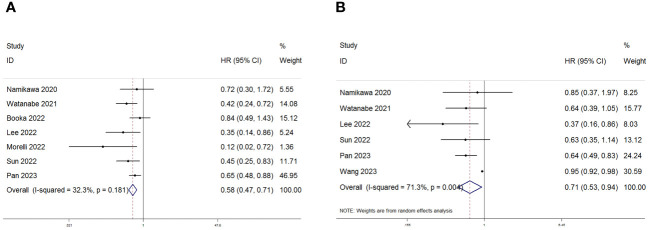
Forest plot for the association between PNI expression and **(A)** overall survival (OS) and **(B)** progression-free survival (PFS) in GC/GEJC patients receiving Immune Checkpoint Inhibitors (ICIs).

Seven studies with a total of 618 patients were analyzed for the correlation between PNI and OS, and the combined results showed that lower PNI was significantly correlated with poorer OS in patients (HR=0.58, 95%CI:0.47–0.71 p=0.001; **Figure 2A**). We also performed a correlation between PNI and PFS analysis, the combined results of 726 cases from 6 studies showed that high PNI before treatment was significantly correlated with better PFS in patients (HR=0.71 95% CI: 0.53–0.94, p=0.004; **Figure 2B**). In order to delve deeper into the prognostic influence of the prognostic nutritional index (PNI) on patients with gastric and gastroesophageal junction (GC/GEJC) cancers undergoing treatment with immune checkpoint inhibitors (ICIs) across various scenarios, and to identify potential sources of heterogeneity, subgroup analyses were conducted based on pre-established stratification criteria, and the results showed that, in terms of OS, both in terms of country (Japan, China, and other), sample size (>100, ≤100), cutoff value (>40, ≤40), and type of analysis (univariate group, multivariate group) Patients with low PNI had worse OS; while the results of subgroup analysis of PFS were not the same as those of OS, PNI did not accurately predict PFS in patients in (Japan, China) countries or with cutoff values <40 or sample sizes higher than 100 (**Tables 3**, **4**).

**Table 3:** Subgroup analysis evaluating the prognostic significance of PNI for OS in GC/GEJC patients treated with ICIs.

Subgroup	NO. of studies	HR (95% CI)	P	Heterogeneity		Model
				I2 (%)	Ph	
Country						
China	2	0.60 (0.46–0.79)	<0.001	9.9	0.292	Fixed
Japan	3	0.62 (0.43–0.87)	0.007	40.6	0.186	Fixed
Other	2	0.28 (0.13–0.63)	0.002	5.9	0.303	Fixed
Cut-off						
≥40	3	0.56 (0.44–0.72)	<0.001	35.2	0.213	Fixed
<40	4	0.61 (0.43–0.87)	0.006	46.8	0.131	Fixed
Sample size						
>100	2	0.59 (0.45–0.76)	<0.001	47.8	0.166	Fixed
<100	5	0.57 (0.41–0.79)	0.001	42.2	0.14	Fixed
Analysis						
Univariate	3	0.65 (0.45–0.94)	0.021	14.7	0.31	Fixed
Multivariate	4	0.55 (0.42–0.70)	<0.001	48.9	0.118	Fixed

**Table 4:** Subgroup analysis evaluating the prognostic significance of PNI for PFS in GC/GEJC patients treated with ICIs.

Subgroup	NO. of studies	HR (95% CI)	P	Heterogeneity		Model
				I2 (%)	Ph	
Country						
China	3	0.76 (0.55–1.07)	0.114	80.7	0.006	Random
Japan	2	0.69 (0.45–1.06)	0.088	0	0.578	Fixed
Other	1	0.37 (0.16–0.86)	0.022	–	–	–
Cut-off						
≥40	4	0.70 (0.49–0.98)	0.039	80.8	0.001	Random
<40	2	0.70 (0.43–1.13)	0.142	0	0.577	Fixed
Sample size						
>100	3	0.76 (0.55–1.06)	0.102	81.7	0.004	Random
<100	3	0.60 (0.39–0.91)	0.016	0	0.377	Fixed
Analysis						
Univariate	3	0.95 (0.92–0.97)	<0.001	0	0.389	Fixed
Multivariate	3	0.62 (0.49–0.77)	<0.001	0	0.468	Fixed

### 3.3 Publication bias and sensitivity analysis

**Figure 3:**
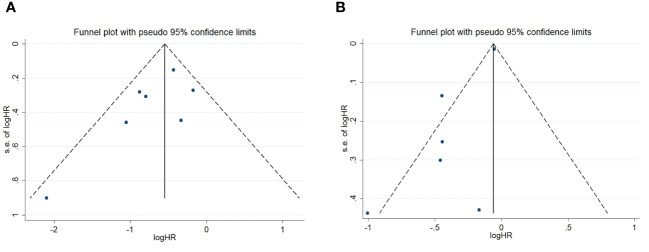
Funnel plots are utilized to assess the presence of publication bias in **(A)** overall survival (OS) and **(B)** progression-free survival (PFS).

**Figure 4:**
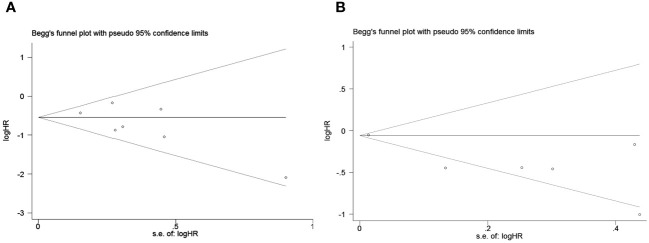
Publication bias test. **(A)** Begg tests for OS; **(B)** Begg tests for PFS.

**Figure 5:**
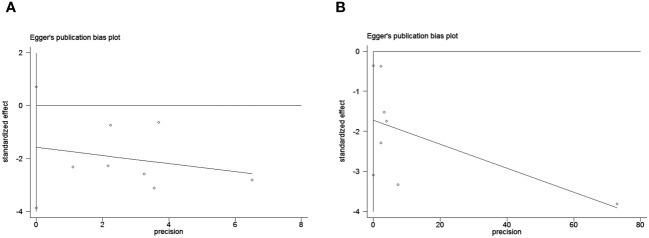
Publication bias test. **(A)** Egger’s test for OS; **(B)** Egger’s test for PFS.

**Figure 6:**
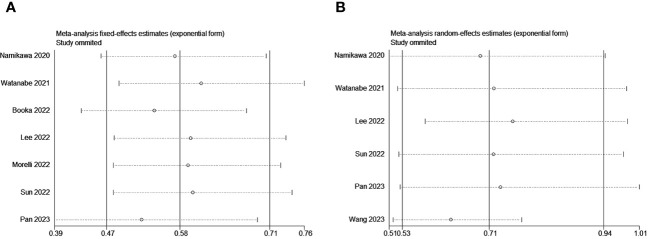
Sensitivity analysis for the pooled results between PNI and **(A)** OS and **(B)** PFS.

To assess potential publication bias, a combination of funnel plots, Begg test, and Egger’s test was employed. The funnel plots for OS exhibited a symmetrical pattern (**Figure 3A**). However, the symmetry of the funnel plot for progression-free survival (PFS) is not as effective as that for overall survival (OS) (**Figure 3B**). The Begg tests indicated no significant publication bias for OS or PFS (OS, p = 0.133; PFS, p = 1.000; **Figure 4**). The outcomes from the Egger (OS, p = 0.135; **Figure 5A**; PFS, p = 0.025; **Figure 5B**) tests revealed a probable publication bias inside the relevant PFS investigations. However, we then conducted a sensitivity analysis, that is, we eliminated each paper in turn and then conducted a summary analysis to see the impact on the final result. The results showed that no single study significantly affected the relationship between PNI and OS and patients’ progression-free survival (PFS)(**Figure 6**). This highlights the reliability of the observed correlation, as the results remain consistent and unaffected by individual studies.

### 3.4 PNI and ORR/DCR association

**Figure 7:**
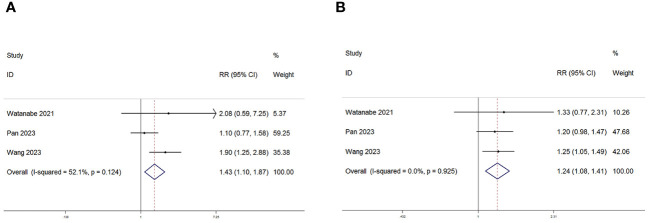
Forest plot for the association between PNI expression and **(A)** Objective response rate (ORR) and **(B)** Disease control rate (DCR) in GC/GEJC patients receiving Immune Checkpoint Inhibitors (ICIs).

Among the eight studies examined, three specifically delved into the relationship between the PNI and the ORR and DCR. Consistently across these three studies, there was a unanimous reporting of a significant statistical correlation between PNI and both DCR and ORR. Subsequent meta-analysis further illuminated the elevated risk ratios (RR) for both ORR (RR=1.43, 95% CI: 1.10–1.87, P=0.008, I2 = 52.1%; **Figure 7A**) and DCR (RR=1.24, 95% CI: 1.08–1.41, P=0.002, I2 = 0%; **Figure 7B**). These results indicate a substantial increase in the likelihood of higher ORR and DCR associated with elevated PNI levels, as supported by the meta-analysis outcomes.

## 4 Discussion

Gastric and gastroesophageal junction (GEJ) cancers are prevalent malignancies of the digestive tract. According to global cancer statistics, their incidence and mortality rates rank among the top five for all cancers (44–46). While the incidence and mortality rates of gastric cancer have been declining, the incidence of GEJ cancer has been gradually increasing (47). In recent years, immune checkpoint inhibitors (ICIs) have achieved significant advancements in cancer treatment, but their efficacy varies significantly among different patients (48). Additionally, there is limited ability to predict the survival outcomes of gastric and GEJ cancer patients treated with ICIs. Therefore, there is an urgent need to identify more effective, simplified, and precise indicators to assist clinicians in predicting post-treatment prognoses. Studies have demonstrated the prognostic significance of the prognostic nutritional index (PNI) as a biomarker in various cancer types (49–51). Nevertheless, the association between the prognostic nutritional index (PNI) and the prognosis of patients with gastric and GEJ cancer undergoing treatment with ICIs has not been conclusively documented, The aim of this meta-analysis is to explore the prognostic significance of PNI in gastric and GEJ cancer patients undergoing ICI therapy. According to our meta-analysis findings, a high prognostic nutritional index (PNI) serves as a favorable prognostic factor for cancer patients undergoing treatment with ICIs. Individuals with elevated PNIs tend to experience extended overall survival (OS) and progression-free survival (PFS) compared to those with lower PNIs. Additionally, they demonstrate increased objective response rates (ORR) and disease control rates (DCR).

As research into immunotherapy deepens, the immunotherapy strategies for gastric and gastroesophageal junction (GEJ) cancers are continually being refined. However, the factors influencing their efficacy remain unclear. Tumor mutational burden (TMB), tumor-infiltrating lymphocytes (TILs), microsatellite instability (MSI), and other tumor biomarkers have been extensively studied as predictive biomarkers for PD-1/L1 inhibitor therapy. However, their application in clinical settings is limited due to the relatively complex detection processes and the lack of consensus on numerical thresholds. The prognostic nutritional index (PNI) is a value derived from nutritional-related serological indicators, such as albumin and lymphocytes. The prognostic nutritional index (PNI) is computed using the formula: PNI = 10 * albumin value (g/dl) + 0.005 * total lymphocyte count in peripheral blood (per mm³).This index can reflect the prognosis of tumor patients (52). Low serum albumin is not only a marker of malnutrition but is also considered a biomarker of systemic inflammation, a view strongly supported by previous research (53). Studies have found that inflammation-related factors can hinder albumin synthesis, and oxidative stress can induce albumin denaturation. Both mechanisms play a crucial role in the rapid decline of serum albumin levels during inflammation (54, 55). In summary, the reduction of serum albumin reflects not only nutritional deficiencies but also a close association with systemic inflammation. Research indicates that serum albumin levels serve as an independent factor influencing the prognosis of patients with malignant tumors (56). Supported by previous research, the interference of inflammation-related factors with albumin synthesis and the denaturation of albumin induced by oxidative stress elucidate the mechanisms behind the rapid decline in serum albumin levels in individuals with inflammation. These insights highlight the multifaceted role of serum albumin as an important biomarker for nutritional status and systemic inflammation processes. Additionally, lymphocytes inhibit the occurrence and development of tumors through cytotoxicity in immune responses (57). Lee YJ’s team conducted a study showing that an elevation in peripheral blood lymphocyte counts before and after treatment correlated with improved progression-free survival (PFS) and overall survival (OS) in non-small cell lung cancer patients undergoing treatment with immune checkpoint inhibitors (ICIs) (58). Moreover, a meta-analysis investigating the relationship between pre-treatment neutrophil-to-lymphocyte ratio (NLR) and clinical outcomes of cancer immunotherapy revealed that a high pre-treatment NLR correlated with unfavorable outcomes of cancer immunotherapy (59).. Recent studies suggest that the activation or proliferation of lymphocytes at early stages can still contribute to improved responses to immunotherapy (60). In summary, it is not difficult to understand why the PNI can reflect the prognosis of tumor patients undergoing immunotherapy.

The objective of this study is to determine whether the prognostic nutritional index (PNI) can serve as a predictor of survival outcomes in patients diagnosed with gastric and gastroesophageal junction (GEJ) cancers undergoing treatment with immune checkpoint inhibitors (ICIs). We performed a comprehensive meta-analysis using data from eight pertinent trials, encompassing 813 patients from four different countries. Our analysis found that a high PNI is significantly associated with better survival rates, indicating that elevated PNI is significantly correlated with improved overall survival (OS) and progression-free survival (PFS) (OS: HR=0.58, 95% CI: 0.47–0.71, p=0.001; PFS: HR=0.71, 95% CI: 0.53–0.94, p=0.004). These results are consistent with most studies, including those by Lee (36) and Pan (43). However, they contradict Booka’s findings, where a retrospective analysis of 30 GEJ cancer patients treated with pembrolizumab or nivolumab showed no significant impact of PNI on OS (P=0.111) and PFS (P=0.381) (38). The discrepancy may be due to the small sample size in Booka’s study, which could introduce bias. Additionally, our subgroup analysis indicated that higher PNI values consistently correlate with better OS regardless of country (China, Japan, Other), sample size (≥100 or <100), cut-off value (>40 or ≤40), and analysis type (multivariate or univariate). Notably, the heterogeneity in PFS might be attributed to differences in country, a cut-off value less than 40, or a sample size greater than 100.To assess potential publication bias, we employed several methods, including funnel plot analysis, Begg’s test, and Egger’s test. Sensitivity analysis and assessment of publication bias further corroborated the steadfastness of the conclusions drawn in this meta-analysis. According to the meta-analysis results, elevated PNI levels significantly increase the likelihood of higher objective response rates (ORR) and disease control rates (DCR). Overall, this meta-analysis has clinical significance, emphasizing the crucial role of optimizing nutritional and immune levels in patients undergoing ICI treatment. The observed associations suggest that by enhancing these factors, the burden of tumors on the body can be alleviated, ultimately improving patient outcomes. Some studies indicate that providing immune-enhancing enteral nutrition is beneficial for improving clinical outcomes and reducing complications in gastric cancer (61, 62). These insights provide a valuable perspective for developing tailored strategies for managing gastric and gastroesophageal junction cancers (GC/GEJC) and integrating ICI treatments. It is important to acknowledge certain limitations when interpreting our findings. Firstly, The sample size in this analysis is relatively limited, with most data originating from Asian countries. Therefore, the value of PNI in European and other populations needs further exploration to determine its applicability across different demographics. Secondly, all included studies in this analysis were retrospective, with inevitable selection biases. Consequently, more high-quality, large-sample, prospective studies are needed in the future to confirm and refine our findings.

## 5 Conclusions

Lower pre-treatment PNI values in patients with gastric and gastroesophageal combined tumors are closely associated with poorer prognosis after patients are treated with ICIs, but further multicenter, prospective, large-data studies are needed to support the study.

## Data availability statement

The original contributions presented in the study are included in the article/supplementary material. Further inquiries can be directed to the corresponding author.

## Author contributions

SH: Conceptualization, Writing – original draft, Writing – review & editing. DS: Investigation, Project administration, Writing – review & editing. RH: Conceptualization, Writing – original draft, Writing – review & editing. LL: Data curation, Formal analysis, Writing – review & editing. YZ: Data curation, Formal analysis, Writing – review & editing. JZ: Supervision, Writing – review & editing.
